# Mutations in the satellite cell gene *MEGF10* cause a recessive congenital myopathy with minicores

**DOI:** 10.1007/s10048-012-0315-z

**Published:** 2012-02-28

**Authors:** Steven E. Boyden, Lane J. Mahoney, Genri Kawahara, Jennifer A. Myers, Satomi Mitsuhashi, Elicia A. Estrella, Anna R. Duncan, Friederike Dey, Elizabeth T. DeChene, Jessica M. Blasko-Goehringer, Carsten G. Bönnemann, Basil T. Darras, Jerry R. Mendell, Hart G. W. Lidov, Ichizo Nishino, Alan H. Beggs, Louis M. Kunkel, Peter B. Kang

**Affiliations:** 1 Division of Genetics, Program in Genomics, and The Manton Center for Orphan Disease Research, Children’s Hospital Boston, Boston, MA USA; 2 Department of Genetics, Harvard Medical School, Boston, MA USA; 3 Department of Neuromuscular Research, National Institute of Neuroscience, National Center of Neurology and Psychiatry, Tokyo, Japan; 4 National Institute of Neurological Disorders and Stroke, National Institutes of Health, Bethesda, MD USA; 5 Department of Neurology, Children’s Hospital Boston and Harvard Medical School, 300 Longwood Avenue, Boston, MA 02115 USA; 6 Center for Gene Therapy Research Institute, Nationwide Children’s Hospital, Columbus, OH USA; 7 Department of Pathology, Children’s Hospital Boston and Harvard Medical School, Boston, MA USA; 8 Department of Pediatrics, Harvard Medical School, Boston, MA USA

**Keywords:** *MEGF10*, Whole genome sequencing, Linkage analysis, Congenital myopathy, Satellite cells, Cleft palate

## Abstract

We ascertained a nuclear family in which three of four siblings were affected with an unclassified autosomal recessive myopathy characterized by severe weakness, respiratory impairment, scoliosis, joint contractures, and an unusual combination of dystrophic and myopathic features on muscle biopsy. Whole genome sequence from one affected subject was filtered using linkage data and variant databases. A single gene, *MEGF10*, contained nonsynonymous mutations that co-segregated with the phenotype. Affected subjects were compound heterozygous for missense mutations c.976T > C (p.C326R) and c.2320T > C (p.C774R). Screening the *MEGF10* open reading frame in 190 patients with genetically unexplained myopathies revealed a heterozygous mutation, c.211C > T (p.R71W), in one additional subject with a similar clinical and histological presentation as the discovery family. All three mutations were absent from at least 645 genotyped unaffected control subjects. *MEGF10* contains 17 atypical epidermal growth factor-like domains, each of which contains eight cysteine residues that likely form disulfide bonds. Both the p.C326R and p.C774R mutations alter one of these residues, which are completely conserved in vertebrates. Previous work showed that murine *Megf10* is required for preserving the undifferentiated, proliferative potential of satellite cells, myogenic precursors that regenerate skeletal muscle in response to injury or disease. Here, knockdown of *megf10* in zebrafish by four different morpholinos resulted in abnormal phenotypes including unhatched eggs, curved tails, impaired motility, and disorganized muscle tissue, corroborating the pathogenicity of the human mutations. Our data establish the importance of *MEGF10* in human skeletal muscle and suggest satellite cell dysfunction as a novel myopathic mechanism.

**Electronic supplementary material:**

The online version of this article (doi:10.1007/s10048-012-0315-z) contains supplementary material, which is available to authorized users.

## Introduction

Congenital myopathies result from a variety of genetic defects [1] that manifest histologically as a disturbance in the structure or organization of myofibers, evident on biopsy by abnormalities such as minicores, central cores, central nuclei, ragged red fibers, nemaline rods, or rimmed vacuoles [2]. By contrast, muscular dystrophies are a class of myopathy in which normally developed muscle tissue undergoes destructive cycles of degeneration and regeneration, leading to distinct pathological hallmarks, including replacement of muscle tissue with fat and infiltration of endomysial connective tissue [3]. These features are usually absent from congenital myopathies, and likewise, minicores and other myopathic disruptions are typically absent from dystrophic muscle. Multiminicore disease (MmD) is an autosomal recessive congenital myopathy characterized by predominantly axial and proximal muscle weakness, frequently in conjunction with progressive scoliosis and respiratory difficulties. The defining histological feature is the presence in a large proportion of muscle fibers of minicores, short regions of sarcomeric disorganization with few or no mitochondria [4]. Mutations in the genes *SEPN1* and *RYR1* explain many but not all cases of MmD [5–8].

The discovery of the genetic basis for such Mendelian diseases has been facilitated by efficient genomewide sequencing methods, notably exome sequencing [9] and whole genome sequencing [10]. Although selective sequencing of exons is currently cost-effective, sequencing of the entire genome offers the opportunity to identify non-exonic variants, extract higher resolution information on copy number and structural mutations, and potentially provide superior coverage of the exome than targeted capture methods achieve. Regardless of the choice of technology, either multiple probands with a genetically homogeneous disease [11] or linkage data [12] are generally required to filter variant lists sufficiently to implicate specific pathogenic mutations. Small families previously considered inaccessible to conventional genetic approaches can now be studied through a combination of linkage analysis and genomewide sequencing.

## Materials and methods

Written informed consent was obtained for all subjects for participation in this study, which was approved by the Institutional Review Board of Children's Hospital Boston (CHB). Zebrafish experiments were conducted in compliance with the guidelines of the CHB Institutional Animal Care and Use Committee. Saliva or blood samples were collected and genomic DNA was isolated. Muscle biopsy slides were reviewed, and for subject 105-1, total RNA was extracted from muscle tissue. All six members of family 1030 were genotyped at 10,204 single nucleotide polymorphisms (SNPs) using the GeneChip Human Mapping 10K 2.0 Xba Array (Affymetrix). Genomewide multipoint parametric linkage scans were performed using MERLIN v1.1.2 [13] with a recessive model, as described previously [14]. Whole genome sequencing and preliminary analysis (initial mappings of reads, local de novo assembly, and variant calling) were performed by Complete Genomics, Inc. using a sequencing-by-ligation method with unchained reads [15]. Polymerase chain reaction (PCR) and Sanger sequencing were performed according to standard protocols. Mutations were genotyped in control subjects using Custom TaqMan SNP Genotyping Assays (Applied Biosystems).

Four custom morpholinos (Gene Tools, LLC) were designed against the intron 4–exon 5 (MO1), intron 5–exon 6 (MO2), exon 8–intron 8 (MO3), and exon 17–intron 17 (MO4) junctions. These morpholinos were intended to induce skipping of the targeted exons, homologous to human exons 7, 8, 10, and 19. MO1 and MO2 were designed against the 3′ splice acceptor side of the intron, and a full exonic skip would induce a frameshift and premature truncation of *megf10*, whereas MO3 and MO4 were designed against the 5′ splice donor side of the intron, and a full exonic skip would induce an in-frame deletion of the exons homologous to those mutated in family 1030. Zebrafish injections, birefringence assays, histochemistry, and immunohistochemistry were performed as described previously [16,17]. For electron microscopy (EM), ultrathin sections were stained with uranyl acetate and lead citrate and were observed with an FEI Tecnai Spirit at 80 kV.

## Results

### Case reports

**Fig. 1:**
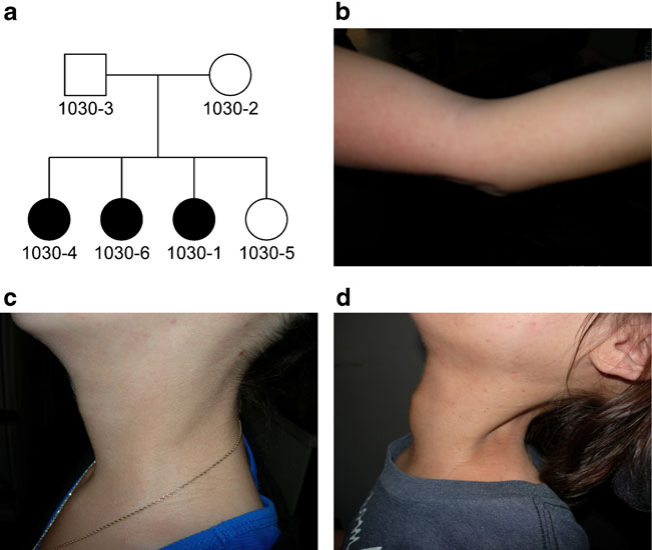
Clinical symptoms of an unclassified autosomal recessive congenital myopathy. **a** Three siblings from a non-consanguineous nuclear family (1030) presented with childhood weakness, respiratory problems, and scoliosis. **b** The arm in subject 1030-1 illustrated elbow contracture to approximately 20°. Biceps and triceps strength in subject 1030-1 were 3+/5 (Medical Research Council scale). **c** The necks of subjects 1030-1 and **d** 1030-6 showed neck atrophy and contractures, more prominent in subject 1030-6. Neck flexion strength was 2/5 in 1030-1 and 0/5 in 1030-6. Neck extension strength was normal in both subjects

**Fig. 2:**
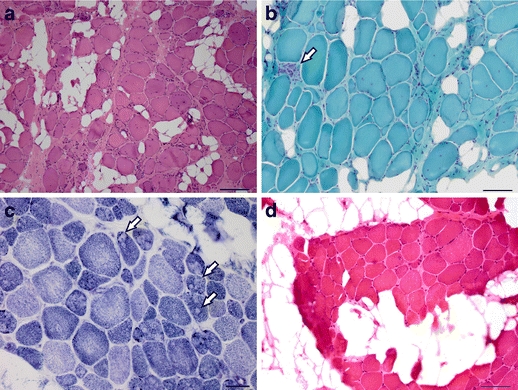
Dystrophic and myopathic pathology in family 1030 muscle biopsy**. a** Hematoxylin and eosin (H&E) staining of sectioned muscle biopsy tissue taken from the left biceps of female subject 1030-1 at age 8 years showed rounded fibers with markedly excessive variation in fiber diameter, fatty replacement, and increased internalized nuclei. Some of the large, hypertrophic fibers were 150 μm in diameter. Inflammation and necrosis were not observed, but scattered basophilic regenerating fibers were present. Magnification is ×10; scale bar is 200 μm. **b** Modified Gomori trichrome staining of 1030-1 biceps highlighted the increased endomysial connective tissue, as well as a single focus of myonecrosis (*arrow*). Magnification is ×20; scale bar is 100 μm. **c** NADH reductase histochemistry of 1030-1 biceps showed many fibers with scattered clearings or “moth-eaten” appearance, reflecting disruption of the myofibril by minicores (*arrows*), predominantly in the darker type I fibers. Magnification is ×40; scale bar is 50 μm. **d** H&E staining of muscle biopsy tissue taken from the right thigh of female subject 1030-4 at age 9 years showed clusters of polygonal myofibers with abundant fatty replacement. There was excessive variation in fiber diameter with frequent hypertrophic fibers, 75–95 μm in diameter. Degeneration, necrosis, or basophilic regenerating fibers were not seen. Magnification is ×20; scale bar is 100 μm

We ascertained a non-consanguineous Caucasian family of mixed European ancestry in which three of four siblings had an undiagnosed autosomal recessive myopathy, which resembled MmD in some respects (Fig. 1 and Online Resource 1). Affected members of family 1030 presented with weakness by 12 months of age, initially in the neck. Weakness was present throughout the body but was primarily axial and proximal and stabilized in adolescence. Facial weakness was also evident. Contractures of the neck were observed in infancy, and contractures of the elbows, knees, and heel cords developed by adulthood. Serum creatine kinase (CK) levels measured prior to age 10 years were mildly elevated. All three patients had childhood onset scoliosis and were treated with spinal fusion surgery, and beginning at age 10–11 years, they all developed respiratory problems treated with bilevel positive airway pressure ventilation at night. One of the patients had cleft palate at birth that was surgically repaired, and a second had a midline ridge on her palate in adulthood, while the palate of the third patient appeared normal. All three affected siblings had hypernasal speech, which was not observed in their unaffected parents and sibling. Muscle biopsy tissue from affected subjects showed replacement of myofibers with fatty tissue, increased perimysial and endomysial connective tissue, and isolated regenerating fibers, features found in muscular dystrophies (Fig. 2). However, moth-eaten fibers and minicores were also observed, suggestive of a congenital myopathy. Rimmed vacuoles, inclusions, central cores, nemaline rods, and ragged red fibers were absent. ATPase staining demonstrated that most atrophic fibers were type I and most hypertrophic fibers were type II, but large type I fibers were also observed. Overall, there was a slight predominance of type I fibers, and they showed a more pronounced variability in size than type II fibers. Prior to any genetic testing, the diagnosis was described as limb girdle muscular dystrophy or spinal muscular atrophy, but the symptoms were also consistent with the clinical spectrum of a congenital myopathy or congenital muscular dystrophy.

Subject 105-1 was a female Caucasian with distant Portuguese ancestry, diagnosed with MmD. No one else in her family was affected. At several weeks of age, she presented with hypotonia, initially manifesting as a weak neck and floppy head. A markedly high arched palate and failure to thrive as a result of poor feeding were noted, as were blue-tinged sclera and hyperflexible joints. She had frequent acute episodes of pulmonary infection as a child but reported no chronic breathing problems and breathed unassisted at night. Serum CK level at age 10 years was mildly elevated at 302 U/L (upper limit of reference range 105 U/L), and mild scoliosis was reported at the same time, which progressed to 20° by age 12 years. Throughout childhood, she continued to exhibit a mild, non-progressive, primarily proximal weakness (including facial weakness), as well as deficits in motor performance. Muscle biopsy showed marked fiber size variation, internalized nuclei, and slightly increased endomysial connective tissue, as well as prominent patches of decreased NADH activity within myofibers, consistent with moth-eaten fibers or minicores (Online Resource 2). Electron microscopy showed focal areas of Z-band disarray devoid of mitochondria and extending across a few sarcomeres in the longitudinal axis, confirming the presence of minicores.

### Linkage analysis

Family 1030 produced positive LOD scores at sixteen genomic loci, ten of which approached the theoretical maximum LOD score of 1.329, and which collectively contained over 1,000 candidate genes (Online Resource 3). Both *SEPN1* and *RYR1* were excluded, as were *ACTA1* and *TPM3*, in which mutations cause congenital fiber type disproportion [18,19], a myopathy clinically similar to MmD. The linkage intervals included the myopathy genes *MYOT* and *CRYAB* [1], but neither of these contained coding or splice site mutations by Sanger sequencing.

### Whole genome sequencing and mutation analysis

Whole genome sequencing of one affected subject from family 1030 yielded a mean coverage depth of 74-fold, with 95.9% of the genome fully called (Online Resource 4). We screened all 16 linked regions for genes containing either homozygous or compound heterozygous variants that altered the amino acid sequence or consensus splice site sequence of an encoded protein. Four linked genes contained putative compound heterozygous, nonsynonymous mutations absent from dbSNP v131. Sanger sequence analysis of three called variants in *ZNF99* showed that all three were homozygous for the reference sequence in all family members; *ZNF99* is a highly repetitive gene and the variant calls may have been due to misaligned reads. Mutations in *LOC100128682* and *LAMA5* were likewise excluded, as they failed to segregate with the phenotype in the entire family. By contrast, the heterozygous missense mutations c.976T > C (p.C326R) and c.2320T > C (p.C774R) in *MEGF10*, the sole remaining candidate, were confirmed by Sanger sequencing to be *in trans* in affected subjects and to segregate with the myopathy in all six family members.

*MEGF10* lies within a linkage peak on chromosome 5 that reached the maximum possible LOD score of 1.329. Both mutations were absent from dbSNP v135, 1,000 Genomes Project data (May 2011 release, 1,094 samples) [20], 5,379 samples from the NHLBI Exome Sequencing Project (ESP) [21] and 56 internal non-myopathy genomes also sequenced at Complete Genomics, Inc. The c.976T > C and c.2320T > C mutations were also absent from 715 and 645 genotyped unaffected control subjects, respectively (Table 1). The p.C326 and p.C774 positions are both completely conserved in 43 species. In accordance with the linkage data, there were no nonsynonymous mutations in *SEPN1*, *RYR1*, *ACTA1*, or *TPM3*. Moreover, none of the more than 70 other genes previously implicated in muscular dystrophies, myopathies, or spinal muscular atrophies [1] contained a homozygous or more than one heterozygous nonsynonymous mutation, once variants in dbSNP v131, 1,000 Genomes, or 56 internal genomes were discarded.

**Table 1:** Missense mutations in *MEGF10* in congenital myopathy patients

Mutation	Amino acid substitution	Family	Exon	Domain	Conservation	Total controls genotyped	Controls with European ancestry
c.211C > T	p.R71W	105	4	Emilin	39/41 species	699	261
c.976T > C	p.C326R	1030	10	EGF-like 6	43/43 species	715	257
c.2320T > C	p.C774R	1030	19	EGF-like 16	43/43 species	645	250

All mutations were heterozygous in affected subjects and absent from all genotyped controls

By Sanger sequencing, we screened the entire open reading frame of *MEGF10* in 190 additional patients with unexplained myopathies, as well as selected exons in another 82 patients. One subject was identified with only a single demonstrable heterozygous mutation; subject 105-1 carried a novel c.211C > T (p.R71W) mutation that was absent from dbSNP v135, 1,000 Genomes data, ESP data, 56 internal genomes, and 699 genotyped unaffected control subjects. The p.R71 residue is conserved in 39 of 41 species (two species of fish having different hydrophilic residues, in contrast to the hydrophobic mutant residue tryptophan). The c.211C > T mutation was heterozygous in an unaffected parent of subject 105-1, so it was not *de novo* dominant. Sanger sequencing further revealed that the subject had no coding or splice site mutations in *SEPN1* or *RYR1*. To search for a second recessive mutation in *MEGF10*, we sequenced its entire open-reading frame in reverse-transcriptase PCR (RT-PCR) amplicons derived from skeletal muscle RNA. Expression of both alleles at the c.211C > T site in exon 4 was verified by amplification and sequencing of full-length cDNA. However, we did not identify a heterozygous exonic copy number change, inversion, or other structural mutation. Nevertheless, because the p.R71W mutation is a nonconservative substitution not found in control subjects, there were no nonsynonymous mutations in *SEPN1* or *RYR1* in this patient, and the clinical and histological characteristics were similar to those of family 1030 patients, subject 105-1 may carry a second mutation in *MEGF10* that we were unable to detect.

### Knockdown of *megf10* in zebrafish

To provide additional evidence for the pathogenicity of *MEGF10* mutations, we knocked down expression of full-length zebrafish *megf10* using four antisense splice-blocking morpholino oligonucleotides (MOs), referred to as MOs 1, 2, 3, and 4 (Online Resource 5). We compared the morphant phenotypes induced by injection of these MOs to uninjected, wild-type fish and fish injected with a standard control morpholino (COMO). The ability of the four MOs to block splicing was verified by performing RT-PCR on total RNA harvested from treated zebrafish (Online Resource 6). All MOs induced the formation of mis-spliced products to some extent, but they generally produced multiple bands not necessarily corresponding in size to the amplicon predicted to result from full skipping of the targeted exon.

**Fig. 3:**
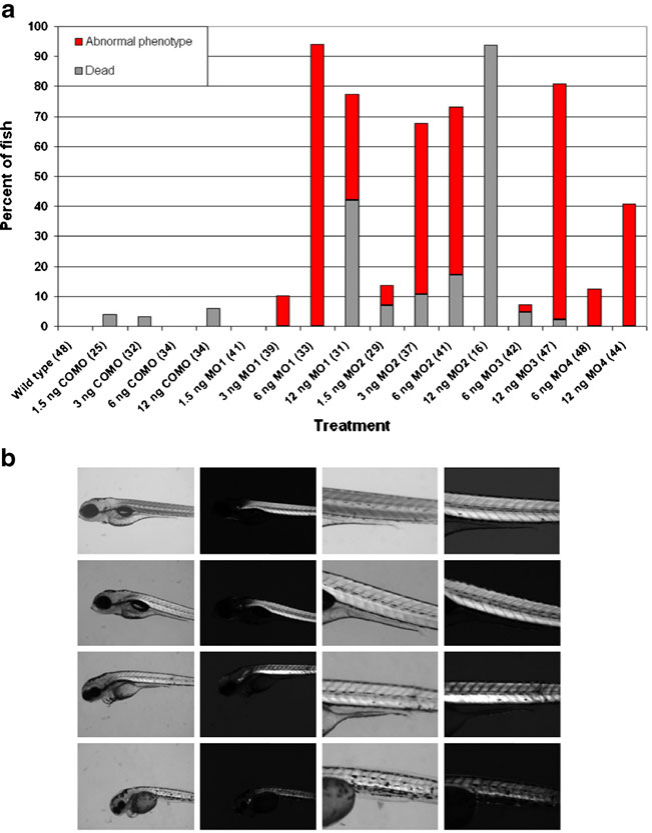
Morphological and birefringence abnormalities in *megf10* morphant zebrafish. **a** Zebrafish treated with anti-*megf10* morpholinos were more likely to die or exhibit an abnormal phenotype than control fish. Abnormal phenotypes were defined as bent or severely curled tails, unhatched eggs, or impaired swimming, including unproductive quivering, rotating in circles, or reduced motility in response to touch. Total numbers of fish examined in each category are given in parentheses after the treatment group label. The remainder of fish in each category appeared unaffected or had non-specific phenotypes, which included four COMO-treated fish (one at each dose) with a slightly curled tail, inflated yolk sac, or bloated appearance, and six fish treated with 6 ng of MO3 that had inflated precardia. **b** Rows from top to bottom are wild-type, 6 ng COMO, 6 ng MO1, and 6 ng MO2. Columns from left to right are brightfield ×5 magnification, birefringence ×5 magnification, brightfield ×11.25 magnification, and birefringence ×11.25 magnification. Compared to control fish, MO1-treated fish showed decreased birefringence, and MO2-treated fish had marked disruption of somite boundaries, a thinner tail with less muscle tissue overall, and substantially decreased birefringence

Anti-*megf10* morpholinos resulted in several phenotypes at 4 days post-fertilization, generally including curled or bent tails, impaired swimming behavior, and diminished motility in response to touch, all consistent with a myopathy (Fig. 3a, Online Resources 7–14). In approximate proportion to dose, MO1-treated fish showed tails bent at the tip and defective swimming. The phenotypes induced by MO2 were the most severe; up to 6 ng of morpholino caused severely curled tails, small heads, ineffective swimming, or unhatched eggs, and at the highest dose of 12 ng, MO2 was lethal. In contrast, 6 ng of MO3 and MO4 produced only a few affected fish out of more than 40 injected, but at 12 ng, both morpholinos produced curved tails, abnormal swimming, and unhatched eggs. Thus, at different doses, all four MOs caused the same basic morphological and motility defects. A single fish in each of the COMO groups showed a mildly curved tail, and two of these fish also had an inflated yolk sac or bloated appearance, which are consistent with injection trauma. Otherwise, neither wild-type nor COMO-treated fish exhibited any phenotypes suggestive of a myopathy.

In morphant fish with severely curled tails, particularly those treated with MO2, the organization of muscle tissue was irregular, with the tail somites showing a less distinct V-shaped chevron pattern than control fish (Fig. 3b). This apparent muscle defect was further investigated using birefringence, the ability of highly organized sarcomeres to rotate polarized light, which can be used to assess muscle integrity [22]. Fish treated with 6 ng of MO1 showed a reduction in birefringence, and 6 ng of MO2 induced a more dramatic decrease in birefringence that corresponded in its location to the disruption of somite structure evident by brightfield microscopy. The abnormal tail morphology and reduced birefringence in *megf10* morphants closely resembled the phenotypes of morphant fish with deficits in known muscular dystrophy gene orthologs [16,23–25].

**Fig. 4:**
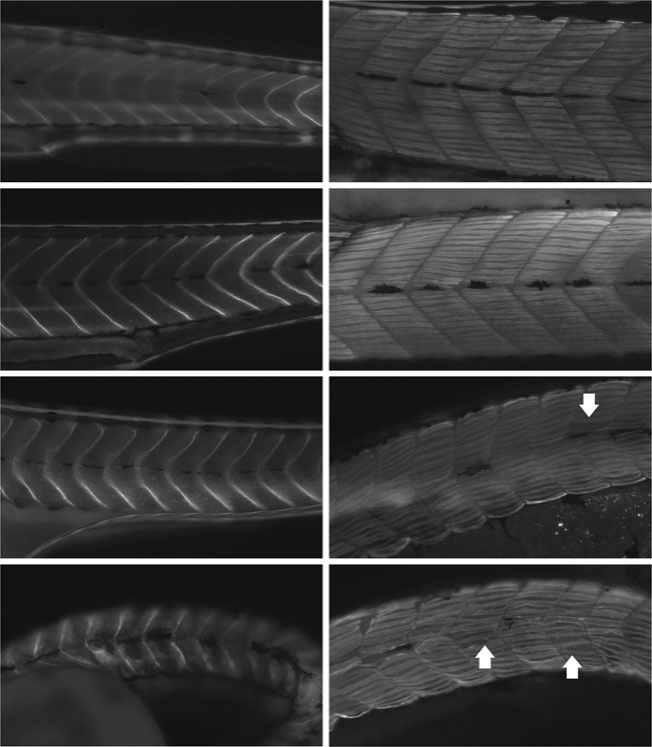
Myopathic features of *megf10* morphant zebrafish muscle**.** Immunohistochemistry was performed with antibodies against β-dystroglycan (*left*) and myosin heavy chain (*right*) on wild-type fish and morphants treated with 6 ng COMO, 6 ng MO1, and 6 ng MO2 (*top to bottom*). Wild-type and COMO-treated fish showed myosepta with straight boundaries and tightly packed myofibers. Compared to control fish, MO1-treated fish showed subtle curving of myosepta and loosely packed myofibers with occasional prominent gaps (*arrow*), and MO2-treated fish showed less distinct somite boundaries and abundant gaps between myofibers (e.g., in somites indicated by *arrows*), creating an overall disorganization of the fiber pattern

To further test whether *megf10* morphant phenotypes reflected a disruption of myofiber integrity, we performed immunohistochemical staining of whole fish with antibodies against β-dystroglycan, a sarcolemmal protein, and myosin heavy chain, a sarcomeric protein. MO1 treatment produced subtle abnormalities in somite shape and abundant gaps between myofibers, while MO2 resulted in a similar but more pronounced disturbance of muscle organization (Fig. 4). The sarcolemmal proteins dystrophin and laminin were also tested and gave similar results as β-dystroglycan (data not shown). To investigate these anomalies in greater detail, we performed toluidine blue staining and EM, which demonstrated that compared to COMO-treated zebrafish, MO2-treated zebrafish muscle showed widespread myofibrillar disorganization and decreased striation (Online Resource 15). These structural defects suggest that the motor impairments of morphant fish were due at least in part to a myopathic process, as opposed to a neuropathy.

## Discussion

*MEGF10* encodes multiple epidermal growth factor (EGF)-like domains 10 (MEGF10), which contains 17 atypical EGF-like domains, each with eight cysteine residues [26]. The p.C326R and p.C774R mutations substitute the fourth and sixth cysteines of EGF-like domains six and sixteen, respectively, for arginines. All eight cysteines in other proteins containing eight-cysteine atypical EGF-like domains form disulfide bridges [27], indicating the mutations in family 1030 likely disrupt MEGF10 protein structure. Similarly, the p.R71W mutation in subject 105-1 is a nonconservative substitution within the emilin domain, which may function in protein–protein interactions [28]. All three mutations were predicted by PolyPhen [29] to be “probably damaging”, and the two cysteine substitutions were predicted to disrupt annotated disulfide bond sites, as expected.

Nonsense and frameshift mutations in *MEGF10* were recently determined to be the cause of a severe congenital myopathy with diaphragmatic weakness, areflexia, and dysphagia. One reported family was compound heterozygous for the c.2320T > C (p.C774R) missense mutation we identified in family 1030 and a frameshift mutation [30]. The phenotypes described therein are generally similar to those of families 1030 and 105, but are more severe. This difference is consistent with the total loss of detectable MEGF10 expression observed in the reported families, compared to the expected misfolding of MEGF10 protein due to disruption of disulfide bonds in family 1030. Accordingly, affected members of family 1030 may retain a small amount of residual MEGF10 function, though genetic background or other factors could also account for clinical heterogeneity between families. Moreover, minicores were not noted in the biopsies from the reported families with null mutations [30,31] but were a prominent feature in our patients with missense mutations. Unstructured minicores in MmD may result from primary defects affecting intracellular redox state or regulation of excitation–contraction coupling at the triad [32]. The molecular mechanisms by which *SEPN1* and *RYR1* mutations result in minicores in MmD have not been elucidated, and likewise, the relationship between *MEGF10* mutations and minicore formation is unclear. Further characterization is required to determine the effect of missense mutations on MEGF10 protein function and sarcomeric structure and to investigate whether *MEGF10* shares a functional pathway with *SEPN1* or *RYR1*.

Human MEGF10 or mouse Megf10 protein has been described as an engulfment receptor expressed in the brain [33–35] and involved in phagocytosis of apoptotic neuronal corpses [36]. One of the patients in family 1030 had overt cleft palate at birth, while a second patient had a midline ridge on her palate and a tendency to reflux liquids into her nose. All three affected subjects had hypernasal speech, which can be a sign of submucous cleft palate [37]. Subject 105-1 had a high-arched palate and poor feeding as an infant. Of seven patients in four reported families with congenital myopathy and mutations in *MEGF10*, one patient had cleft palate and an additional three patients had high-arched palate [30]. Although these clinical features are relatively nonspecific, occurring commonly in patients with myopathic facies, the expression of mouse *Megf10* in brain [35,38] is consistent with a role in craniofacial development. In addition, Megf10 interacts with Ap2m1, one of four subunits of the assembly protein complex 2 (AP2) [35]. Ablation of *Ap2b1*, which encodes another of the AP2 subunits, causes cleft palate in mouse [39]. Furthermore, *Megf10* in muscle is only expressed in cells that also express *Pax7* [38], and *Pax7* ^−/−^ null mice exhibit facial malformations of the maxilla and nose due to a defect in cephalic neural crest development [40]. Lastly, some morphant zebrafish treated with MO1 or MO2 showed small heads relative to control fish, further suggesting a possible role for *megf10* in head development. Collectively, these data indicate that *MEGF10* may influence palate formation or facial midline determination in humans, but more research is required to test this hypothesis.

In addition to brain, mouse *Megf10* is expressed in muscle satellite cells [38,41], a population of committed, myogenic precursors that specifically express the transcription factor Pax7 and contribute to the regeneration of adult muscle in response to exercise, disease, or injury [42,43]. The importance of satellite cell function in mouse muscle maintenance has been demonstrated; *Pax7* ^−/−^ mice have a reduced number of satellite cells as embryos and very few or none as adults, and muscle regeneration is impaired in these mice [44]. Recent studies further confirmed that Pax7^+^ satellite cells are completely required for muscle regeneration [45–47], suggesting that in humans, compromised satellite cell function could result in a myopathic phenotype. Expression of *Megf10* in skeletal muscle is restricted to satellite cells and is low in resting muscle but increases dramatically in regenerating muscle upon activation of satellite cells by treatment with cardiotoxin [38]. *Megf10* regulates the differentiation program in skeletal myoblasts, as overexpression of *Megf10* in C2C12 myoblasts increases proliferation and decreases differentiation, whereas knockdown of *Megf10* in primary myoblasts and individual myofibers causes precocious differentiation and a reduction of activated satellite cells [38].

Consistent with the role of *Megf10* in mouse myoblasts, our data establish that human *MEGF10* is required for normal muscle function. Due to presumed disruption of disulfide bonds, the p.C326R and p.C774R mutations in family 1030 likely result in a loss of *MEGF10* function, in which case we hypothesize that excessive differentiation of satellite cells could deplete the population of self-renewing cells available to regenerate damaged muscle fibers. Such impairment could potentially explain the dystrophic features observed in patient biopsy tissue at ages 8 and 9. Notably, a recent study found that *Sepn1* ^−/−^ null mice showed a decrease in the baseline number of satellite cells in adult skeletal muscle, as well as imperfect muscle regeneration in response to a first injury and an inability to regenerate muscle following a second injury. MmD patients with *SEPN1* deficiency also had a reduction in their satellite cell population [48]. These data suggest that both MmD and the myopathy described here, which share some clinical and histological characteristics, may arise through a common pathogenic mechanism of satellite cell dysfunction.

## Electronic supplementary material

Below is the link to the electronic supplementary material.

Online Resource 1 (PDF 19 kb)

Online Resource 2. **Myopathic pathology in subject 105–1 muscle biopsy** **a** Hematoxylin and eosin staining of biopsy tissue taken from left quadriceps at age 10 showed excessive variation of fiber diameters and increased internalized nuclei. A single degenerating fiber was present (*arrow*). Necrosis, regeneration, fibrosis, and fatty replacement were not observed. Magnification is ×20; scale bar is 50 μm. **b** NADH reductase histochemistry showed a “moth-eaten” appearance in fibers of both fiber types, indicative of minicores. Magnification is ×40; *scale bar* is 20 μm. **c** Ultrastructural image showed the sharply demarcated area of a minicore, with disruption of the sarcomeric organization and myofibrils. There was focal dissolution of Z-bands and thick filaments over several sarcomeres. Magnification is ×2,500; scale bar is 2 μm (courtesy of Howard Mulhern) (PDF 556 kb)

Online Resource 3 (PDF 12 kb)

Online Resource 4 (PDF 11 kb)

Online Resource 5 (PDF 8 kb)

Online Resource 6. **Effect on splicing of anti-** ***megf10*** **morpholinos** Agarose gel electrophoresis of RT-PCR products derived from MO-treated zebrafish shows numerous products absent from wild-type (W) and COMO-treated (C) fish. The *upper lane* *marker* indicates the morpholino number or control group, the *lower lane* *marker* indicates the dose (ng), and the ladder marker indicates the fragment size in base pairs. Lanes 2–13 and 15 contain amplicons spanning exons 4–7, lanes 16–18 contain amplicons spanning exons 7–9, and lanes 19–21 contain amplicons spanning exons 16–18. For the first lane with each morpholino, arrows indicate the size of the band expected from a full exonic skip. Mis-spliced products may appear reduced in intensity relative to the main product due to nonsense-mediated mRNA decay (PDF 69 kb)

Online Resource 7. **Normal movement of uninjected wild-type zebrafish at 4 days post-fertilization** Uninjected wild-type zebrafish had straight tails and rapidly swam away in a straight trajectory when touched. (MP4 116 kb)

Online Resource 8. **Normal movement of COMO-treated morphant zebrafish at 4 days post-fertilization** COMO-treated zebrafish had straight tails and rapidly swam away in a straight trajectory when touched. (MP4 129 kb)

Online Resource 9. **Impaired movement of** ***megf10*** **morphant zebrafish at 4 days post-fertilization** A morphant zebrafish treated with 3 ng of MO1 had a slightly bent tail tip and did not initially respond to touch, but was later able to swim away. (MP4 249 kb)

Online Resource 10. **Impaired movement of** ***megf10*** **morphant zebrafish at 4 days post-fertilization** A morphant zebrafish treated with 6 ng of MO1 had a gently curved tail and spastic tail movements that turned the fish in circles but were ineffective for locomotion. (MP4 202 kb)

Online Resource 11. **Impaired movement of** ***megf10*** **morphant zebrafish at 4 days post-fertilization** A morphant zebrafish treated with 3 ng of MO2 had a severely curved tail and displayed an unproductive quivering motion, capable only of rotating the fish, either autonomously or in response to touch. (MP4 235 kb)

Online Resource 12. **Impaired movement of** ***megf10*** **morphant zebrafish at 4 days post-fertilization** A morphant zebrafish treated with 6 ng of MO2 had a severely curved tail and displayed an unproductive quivering motion, capable only of rotating the fish. (MP4 218 kb)

Online Resource 13. **Impaired movement of** ***megf10*** **morphant zebrafish at 4 days post-fertilization** A collection of fish treated with 12 ng of MO3 showed unhatched eggs, varying degrees of tail curvature, and a range of response to touch, from normal linear swimming to unproductive, rapid undulation of the tail resulting only in spinning in place. Heartbeats were observed in all fish to verify they were alive. (MP4 122 kb)

Online Resource 14. **Impaired movement of** ***megf10*** **morphant zebrafish at 4 days post-fertilization** A collection of fish treated with 12 ng of MO4 show moderately curved tails and very limited response to touch. Heartbeats were observed in all fish to verify they were alive. (MP4 283 kb)

Online Resource 15. **Myopathic features of** ***megf10*** **morphant zebrafish myofibrils** **a** Epon-embedded semi-thin muscle sections from zebrafish injected with 6 ng of COMO were stained with toluidine blue and observed by light microscopy at 4 days post-fertilization to have striated, elongated fibers. **b** Similarly prepared zebrafish injected with 6 ng of MO2 showed fewer striated fibers than COMO-injected fish, and several round fibers with prominent nuclei. Scale bar for **a** and **b** is 20 μm. **c** Electron microscopy (EM) of ultra-thin sections showed patterned myofibrils in COMO-injected zebrafish muscle at 4 days post-fertilization. **d** In EM of MO2-injected zebrafish muscle, myofibrils were largely disorganized. Scale bar for **c** and **d** is 0.5 μm. (PDF 158 kb)

## References

[1] Kaplan JC (2010). The 2011 version of the gene table of neuromuscular disorders. Neuromuscul Disord.

[2] Sewry CA (2008). Pathological defects in congenital myopathies. J Muscle Res Cell Motil.

[3] Dalkilic I, Kunkel LM (2003). Muscular dystrophies: genes to pathogenesis. Curr Opin Genet Dev.

[4] Engel AG, Gomez MR, Groover RV (1971). Multicore disease. A recently recognized congenital myopathy associated with multifocal degeneration of muscle fibers. Mayo Clin Proc.

[5] Moghadaszadeh B, Petit N, Jaillard C, Brockington M, Roy SQ, Merlini L, Romero N, Estournet B, Desguerre I, Chaigne D, Muntoni F, Topaloglu H, Guicheney P (2001). Mutations in SEPN1 cause congenital muscular dystrophy with spinal rigidity and restrictive respiratory syndrome. Nat Genet.

[6] Ferreiro A, Quijano-Roy S, Pichereau C, Moghadaszadeh B, Goemans N, Bonnemann C, Jungbluth H, Straub V, Villanova M, Leroy JP, Romero NB, Martin JJ, Muntoni F, Voit T, Estournet B, Richard P, Fardeau M, Guicheney P (2002). Mutations of the selenoprotein N gene, which is implicated in rigid spine muscular dystrophy, cause the classical phenotype of multiminicore disease: reassessing the nosology of early-onset myopathies. Am J Hum Genet.

[7] Ferreiro A, Monnier N, Romero NB, Leroy JP, Bonnemann C, Haenggeli CA, Straub V, Voss WD, Nivoche Y, Jungbluth H, Lemainque A, Voit T, Lunardi J, Fardeau M, Guicheney P (2002). A recessive form of central core disease, transiently presenting as multi-minicore disease, is associated with a homozygous mutation in the ryanodine receptor type 1 gene. Ann Neurol.

[8] Monnier N, Ferreiro A, Marty I, Labarre-Vila A, Mezin P, Lunardi J (2003). A homozygous splicing mutation causing a depletion of skeletal muscle RYR1 is associated with multi-minicore disease congenital myopathy with ophthalmoplegia. Hum Mol Genet.

[9] Ng SB, Buckingham KJ, Lee C, Bigham AW, Tabor HK, Dent KM, Huff CD, Shannon PT, Jabs EW, Nickerson DA, Shendure J, Bamshad MJ (2010). Exome sequencing identifies the cause of a Mendelian disorder. Nat Genet.

[10] Rios J, Stein E, Shendure J, Hobbs HH, Cohen JC (2010). Identification by whole-genome resequencing of gene defect responsible for severe hypercholesterolemia. Hum Mol Genet.

[11] Ng SB, Bigham AW, Buckingham KJ, Hannibal MC, McMillin MJ, Gildersleeve HI, Beck AE, Tabor HK, Cooper GM, Mefford HC, Lee C, Turner EH, Smith JD, Rieder MJ, Yoshiura K, Matsumoto N, Ohta T, Niikawa N, Nickerson DA, Bamshad MJ, Shendure J (2010). Exome sequencing identifies MLL2 mutations as a cause of Kabuki syndrome. Nat Genet.

[12] Bilguvar K, Ozturk AK, Louvi A, Kwan KY, Choi M, Tatli B, Yalnizoglu D, Tuysuz B, Caglayan AO, Gokben S, Kaymakcalan H, Barak T, Bakircioglu M, Yasuno K, Ho W, Sanders S, Zhu Y, Yilmaz S, Dincer A, Johnson MH, Bronen RA, Kocer N, Per H, Mane S, Pamir MN, Yalcinkaya C, Kumandas S, Topcu M, Ozmen M, Sestan N, Lifton RP, State MW, Gunel M (2010). Whole-exome sequencing identifies recessive WDR62 mutations in severe brain malformations. Nature.

[13] Abecasis GR, Cherny SS, Cookson WO, Cardon LR (2002). Merlin-rapid analysis of dense genetic maps using sparse gene flow trees. Nat Genet.

[14] Boyden SE, Salih MA, Duncan AR, White AJ, Estrella EA, Burgess SL, Seidahmed MZ, Al-Jarallah AS, Alkhalidi HM, Al-Maneea WM, Bennett RR, Alshemmari SH, Kunkel LM, Kang PB (2010). Efficient identification of novel mutations in patients with limb girdle muscular dystrophy. Neurogenetics.

[15] Drmanac R, Sparks AB, Callow MJ, Halpern AL, Burns NL, Kermani BG, Carnevali P, Nazarenko I, Nilsen GB, Yeung G, Dahl F, Fernandez A, Staker B, Pant KP, Baccash J, Borcherding AP, Brownley A, Cedeno R, Chen L, Chernikoff D, Cheung A, Chirita R, Curson B, Ebert JC, Hacker CR, Hartlage R, Hauser B, Huang S, Jiang Y, Karpinchyk V, Koenig M, Kong C, Landers T, Le C, Liu J, McBride CE, Morenzoni M, Morey RE, Mutch K, Perazich H, Perry K, Peters BA, Peterson J, Pethiyagoda CL, Pothuraju K, Richter C, Rosenbaum AM, Roy S, Shafto J, Sharanhovich U, Shannon KW, Sheppy CG, Sun M, Thakuria JV, Tran A, Vu D, Zaranek AW, Wu X, Drmanac S, Oliphant AR, Banyai WC, Martin B, Ballinger DG, Church GM, Reid CA (2010). Human genome sequencing using unchained base reads on self-assembling DNA nanoarrays. Science.

[16] Kawahara G, Guyon JR, Nakamura Y, Kunkel LM (2010). Zebrafish models for human FKRP muscular dystrophies. Hum Mol Genet.

[17] Mitsuhashi S, Hatakeyama H, Karahashi M, Koumura T, Nonaka I, Hayashi YK, Noguchi S, Sher RB, Nakagawa Y, Manfredi G, Goto Y, Cox GA, Nishino I (2011). Muscle choline kinase beta defect causes mitochondrial dysfunction and increased mitophagy. Hum Mol Genet.

[18] Laing NG, Clarke NF, Dye DE, Liyanage K, Walker KR, Kobayashi Y, Shimakawa S, Hagiwara T, Ouvrier R, Sparrow JC, Nishino I, North KN, Nonaka I (2004). Actin mutations are one cause of congenital fibre type disproportion. Ann Neurol.

[19] Clarke NF, Kolski H, Dye DE, Lim E, Smith RL, Patel R, Fahey MC, Bellance R, Romero NB, Johnson ES, Labarre-Vila A, Monnier N, Laing NG, North KN (2008). Mutations in TPM3 are a common cause of congenital fiber type disproportion. Ann Neurol.

[20] (2010) A map of human genome variation from population-scale sequencing. Nature 467:1061–1073 10.1038/nature09534 PMC3042601 20981092

[21] Exome Variant Server. NHLBI Exome Sequencing Project (ESP). http://evs.gs.washington.edu/EVS/. Accessed October 2011

[22] Granato M, van Eeden FJ, Schach U, Trowe T, Brand M, Furutani-Seiki M, Haffter P, Hammerschmidt M, Heisenberg CP, Jiang YJ, Kane DA, Kelsh RN, Mullins MC, Odenthal J, Nusslein-Volhard C (1996). Genes controlling and mediating locomotion behavior of the zebrafish embryo and larva. Development.

[23] Guyon JR, Mosley AN, Zhou Y, O'Brien KF, Sheng X, Chiang K, Davidson AJ, Volinski JM, Zon LI, Kunkel LM (2003). The dystrophin associated protein complex in zebrafish. Hum Mol Genet.

[24] Guyon JR, Mosley AN, Jun SJ, Montanaro F, Steffen LS, Zhou Y, Nigro V, Zon LI, Kunkel LM (2005). Delta-sarcoglycan is required for early zebrafish muscle organization. Exp Cell Res.

[25] Nixon SJ, Wegner J, Ferguson C, Mery PF, Hancock JF, Currie PD, Key B, Westerfield M, Parton RG (2005). Zebrafish as a model for caveolin-associated muscle disease; caveolin-3 is required for myofibril organization and muscle cell patterning. Hum Mol Genet.

[26] Nagase T, Nakayama M, Nakajima D, Kikuno R, Ohara O (2001). Prediction of the coding sequences of unidentified human genes. XX. The complete sequences of 100 new cDNA clones from brain which code for large proteins in vitro. DNA Res.

[27] Wouters MA, Rigoutsos I, Chu CK, Feng LL, Sparrow DB, Dunwoodie SL (2005). Evolution of distinct EGF domains with specific functions. Protein Sci.

[28] Callebaut I, Mignotte V, Souchet M, Mornon JP (2003). EMI domains are widespread and reveal the probable orthologs of the *Caenorhabditis elegans* CED-1 protein. Biochem Biophys Res Commun.

[29] Ramensky V, Bork P, Sunyaev S (2002). Human non-synonymous SNPs: server and survey. Nucleic Acids Res.

[30] Logan CV, Lucke B, Pottinger C, Abdelhamed ZA, Parry DA, Szymanska K, Diggle CP, Riesen A, Morgan JE, Markham G, Ellis I, Manzur AY, Markham AF, Shires M, Helliwell T, Scoto M, Hubner C, Bonthron DT, Taylor GR, Sheridan E, Muntoni F, Carr IM, Schuelke M, Johnson CA (2011). Mutations in MEGF10, a regulator of satellite cell myogenesis, cause early onset myopathy, areflexia, respiratory distress and dysphagia (EMARDD). Nat Genet.

[31] Hartley L, Kinali M, Knight R, Mercuri E, Hubner C, Bertini E, Manzur AY, Jimenez-Mallebrera C, Sewry CA, Muntoni F (2007). A congenital myopathy with diaphragmatic weakness not linked to the SMARD1 locus. Neuromuscul Disord.

[32] Treves S, Jungbluth H, Muntoni F, Zorzato F (2008). Congenital muscle disorders with cores: the ryanodine receptor calcium channel paradigm. Curr Opin Pharmacol.

[33] Hamon Y, Trompier D, Ma Z, Venegas V, Pophillat M, Mignotte V, Zhou Z, Chimini G (2006). Cooperation between engulfment receptors: the case of ABCA1 and MEGF10. PLoS One.

[34] Suzuki E, Nakayama M (2007). The mammalian Ced-1 ortholog MEGF10/KIAA1780 displays a novel adhesion pattern. Exp Cell Res.

[35] Suzuki E, Nakayama M (2007). MEGF10 is a mammalian ortholog of CED-1 that interacts with clathrin assembly protein complex 2 medium chain and induces large vacuole formation. Exp Cell Res.

[36] Wu HH, Bellmunt E, Scheib JL, Venegas V, Burkert C, Reichardt LF, Zhou Z, Farinas I, Carter BD (2009). Glial precursors clear sensory neuron corpses during development via Jedi-1, an engulfment receptor. Nat Neurosci.

[37] Moss AL, Piggott RW, Jones KJ (1988). Submucous cleft palate. BMJ.

[38] Holterman CE, Le Grand F, Kuang S, Seale P, Rudnicki MA (2007). Megf10 regulates the progression of the satellite cell myogenic program. J Cell Biol.

[39] Li W, Puertollano R, Bonifacino JS, Overbeek PA, Everett ET (2010) Disruption of the Murine Ap2beta1 Gene Causes Nonsyndromic Cleft Palate. Cleft Palate Craniofac J 47:566–573 10.1597/09-145 PMC3691559 20500056

[40] Mansouri A, Stoykova A, Torres M, Gruss P (1996). Dysgenesis of cephalic neural crest derivatives in Pax7−/− mutant mice. Development.

[41] Seale P, Ishibashi J, Holterman C, Rudnicki MA (2004). Muscle satellite cell-specific genes identified by genetic profiling of MyoD-deficient myogenic cell. Dev Biol.

[42] Seale P, Sabourin LA, Girgis-Gabardo A, Mansouri A, Gruss P, Rudnicki MA (2000). Pax7 is required for the specification of myogenic satellite cells. Cell.

[43] Peault B, Rudnicki M, Torrente Y, Cossu G, Tremblay JP, Partridge T, Gussoni E, Kunkel LM, Huard J (2007). Stem and progenitor cells in skeletal muscle development, maintenance, and therapy. Mol Ther.

[44] Oustanina S, Hause G, Braun T (2004). Pax7 directs postnatal renewal and propagation of myogenic satellite cells but not their specification. EMBO J.

[45] Murphy MM, Lawson JA, Mathew SJ, Hutcheson DA, Kardon G (2011). Satellite cells, connective tissue fibroblasts and their interactions are crucial for muscle regeneration. Development.

[46] Lepper C, Partridge TA, Fan CM (2011). An absolute requirement for Pax7-positive satellite cells in acute injury-induced skeletal muscle regeneration. Development.

[47] Sambasivan R, Yao R, Kissenpfennig A, Van Wittenberghe L, Paldi A, Gayraud-Morel B, Guenou H, Malissen B, Tajbakhsh S, Galy A (2011). Pax7-expressing satellite cells are indispensable for adult skeletal muscle regeneration. Development.

[48] Castets P, Bertrand AT, Beuvin M, Ferry A, Le Grand F, Castets M, Chazot G, Rederstorff M, Krol A, Lescure A, Romero NB, Guicheney P, Allamand V (2011). Satellite cell loss and impaired muscle regeneration in selenoprotein N deficiency. Hum Mol Genet.

