# Supplementary material for: Mutations in the satellite cell gene MEGF10 cause a recessive congenital myopathy with minicores
Source: Neurogenetics. 2012 Feb 28;13(2):115–24. doi: 10.1007/s10048-012-0315-z (PMC3332380; doi:10.1007/s10048-012-0315-z)
Supplement: Supplementary file 1 — (PDF 19 kb) [file 10048_2012_315_MOESM1_ESM.pdf]

Online Resource 1. Clinical histories and results of physical examinations of affected members of family 1030

Subject	**1030-4**	**1030-6**	**1030-1**
Gender	Female	Female	Female
Handedness	Right	Right	Right
**History**			
Symptoms as infant	Hypotonia, poor suck, cleft palate, neck flexor weakness, neck contracture	Neck flexor weakness, neck contracture, febrile seizures at 8 to 23 mos.	Neck flexor weakness, neck contracture
Symptoms as child	Frequent falling, difficulty running, obesity	Frequent falling, slow running, extremity weakness	Tendency to fall hard on knees, difficulty ascending stairs
Weakness progression	Increased at menarche, then static	Increased at menarche, then static	Increased at menarche, then static
Serum creatine kinase	Normal or slightly elevated	174, 217 U/L (reference 37-173)	Slightly elevated
Age at onset of scoliosis	Unknown, before 13 yrs.	7-8 yrs.	9 yrs.
Scoliosis	72 degrees	34 degrees	32 degrees
Back surgery	14 yrs., fused vertebrae	11 yrs., Harrington rods placed	10 yrs., hooks and Harrington rods placed
Age at loss of ambulation	15 yrs.	11 yrs., after surgery	Still ambulatory at 23 yrs.
Age at onset of respiratory problems	11 yrs. (bronchitis and pneumonia)	11 yrs.	10 yrs.
Respiratory involvement	Chronic restrictive lung disease	Chronic restrictive lung disease	Chronic restrictive lung disease
Respiratory treatment	BiPAP at night, supplemental oxygen	BiPAP at night	BiPAP at night
Cardiac involvement	None	None	None
Other	Acid reflux, head MRI at 12 yrs. showed atrophy of cortical gray matter	Acid reflux, reflux into nose on Barium swallow test, dysphagia with large pills	Dysphagia with pills, episodes of choking, weakness at upper lip
**Neuromuscular exam**			
Age at examination	28 yrs.	25 yrs.	23 yrs.
Cognitive status	Normal, attends college	Normal, attends college	Normal, attends college
Cleft palate	Yes (scar from surgical repair at 3 yrs.)	Midline ridge	No
Hypernasal voice	Yes	Yes	Yes
Pupils	Equal, round, reactive	Equal, round, reactive	Equal, round, reactive
Reflexes	Mostly absent	Mostly absent	Mostly absent
Extraocular movements	Intact without nystagmyus	Intact without nystagmyus	Intact without nystagmyus
Facial weakness	Diffuse, moderate	Diffuse, moderate	Diffuse, moderate
Neck contractures	Yes	Yes	Yes
Elbow contractures	10 degrees	30 degrees	20 degrees
Metacarpophalangeal contractures	NA	Yes (right > left)	NA
Knee contractures	20 degrees	10/0 degrees (R/L)	5 degrees
Heel cord contractures	-5 degrees	30/20 degrees (R/L), surgery at 12 yrs.	-10/-5 degrees (R/L)

Subject	**1030-4**	**1030-6**	**1030-1**
Neck flexion	2	0	2
Neck extension	5	5	5
First dorsal interosseous	4	1/2 (R/L)	3
Abductor digiti minimi	4	1/2 (R/L)	3
Abductor pollicis brevis	4	2	3
Extensor digitorum communis	4	0/3- (R/L)	3
Grip	4	3	3
Wrist extension	4	0/3 (R/L)	4
Wrist flexion	5	5/4 (R/L)	5
Biceps	4	3	3+
Triceps	4	2	3+
Deltoids	4	3-	3+
Extensor hallucis longus	0	2	3
Tibialis anterior	3	2	3
Peroneus longus and brevis	3	2	3
Gastrocnemius	5	5	5
Tibialis posterior	5	5	5
Quadriceps	5/4- (R/L)	3	5
Hamstrings	4	3	3
Iliopsoas (sitting up)	4	2	3

Strength tests are out of a maximum of 5, on the Medical Research Council scale. Abbreviations are as follows: mos., months; yrs., years; BiPAP, bilevel positive airway pressure ventilation; MRI, magnetic resonance imaging; R/L, right/left.
