# Supplementary figures and images for: Mutations in the satellite cell gene MEGF10 cause a recessive congenital myopathy with minicores
Source: Neurogenetics. 2012 Feb 28;13(2):115–24. doi: 10.1007/s10048-012-0315-z (PMC3332380; doi:10.1007/s10048-012-0315-z)

**a**

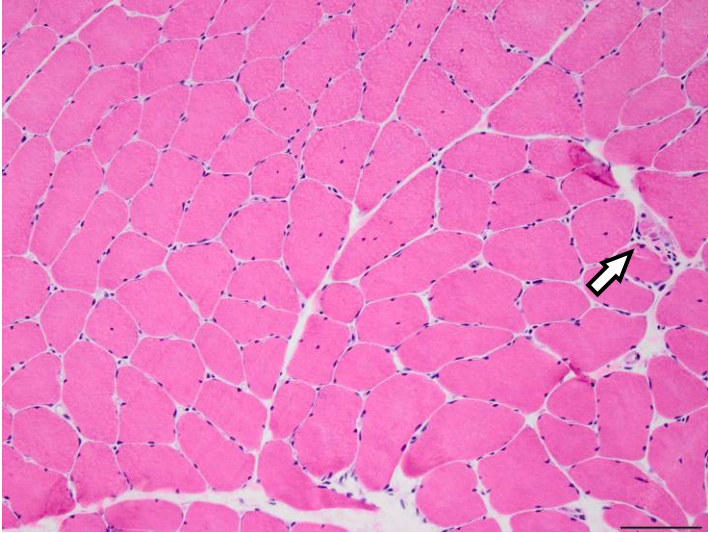

**b**

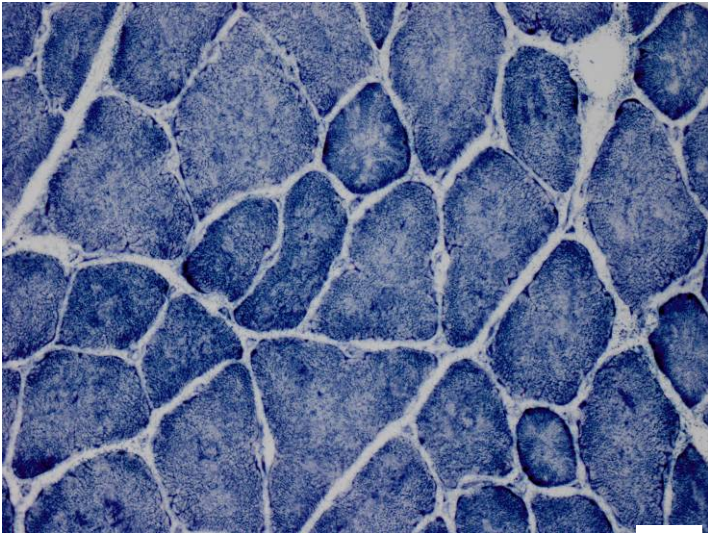

**c**

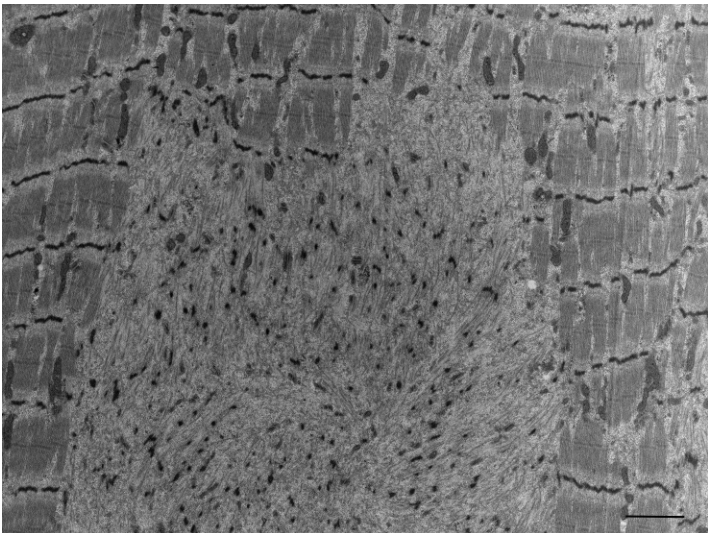

Supplement: Supplementary file 2 — Myopathic pathology in subject 105–1 muscle biopsy a Hematoxylin and eosin staining of biopsy tissue taken from left quadriceps at age 10 showed excessive variation of fiber diameters and increased internalized nuclei. A single degenerating fiber was present (arrow). Necrosis, regeneration, fibrosis, and fatty replacement were not observed. Magnification is ×20; scale bar is 50 μm. b NADH reductase histochemistry showed a “moth-eaten” appearance in fibers of both fiber types, indicative of minicores. Magnification is ×40; scale bar is 20 μm. c Ultrastructural image showed the sharply demarcated area of a minicore, with disruption of the sarcomeric organization and myofibrils. There was focal dissolution of Z-bands and thick filaments over several sarcomeres. Magnification is ×2,500; scale bar is 2 μm (courtesy of Howard Mulhern) (PDF 556 kb) [file 10048_2012_315_MOESM2_ESM.pdf]

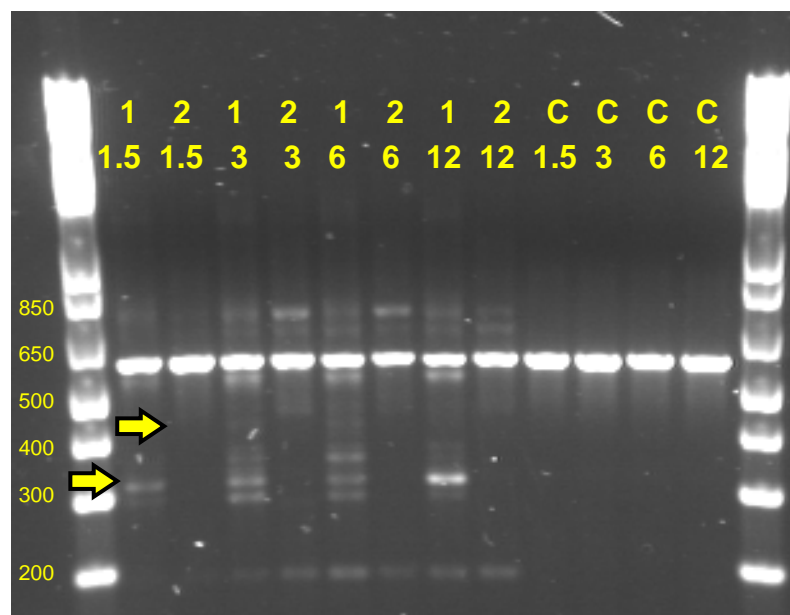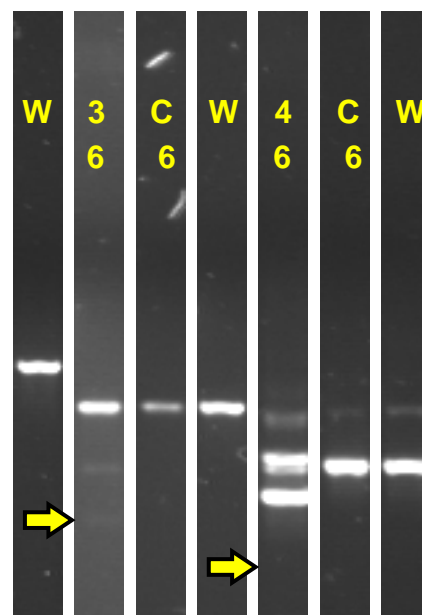

Supplement: Supplementary file 6 — Effect on splicing of anti- megf10 morpholinos Agarose gel electrophoresis of RT-PCR products derived from MO-treated zebrafish shows numerous products absent from wild-type (W) and COMO-treated (C) fish. The upper lane marker indicates the morpholino number or control group, the lower lane marker indicates the dose (ng), and the ladder marker indicates the fragment size in base pairs. Lanes 2–13 and 15 contain amplicons spanning exons 4–7, lanes 16–18 contain amplicons spanning exons 7–9, and lanes 19–21 contain amplicons spanning exons 16–18. For the first lane with each morpholino, arrows indicate the size of the band expected from a full exonic skip. Mis-spliced products may appear reduced in intensity relative to the main product due to nonsense-mediated mRNA decay (PDF 69 kb) [file 10048_2012_315_MOESM6_ESM.pdf]

**a**

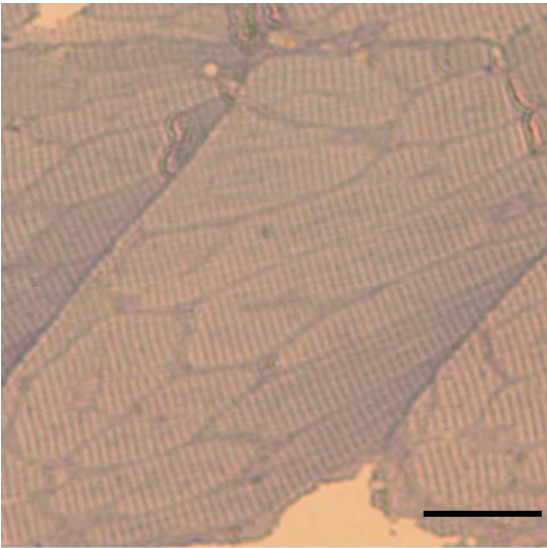

**b**

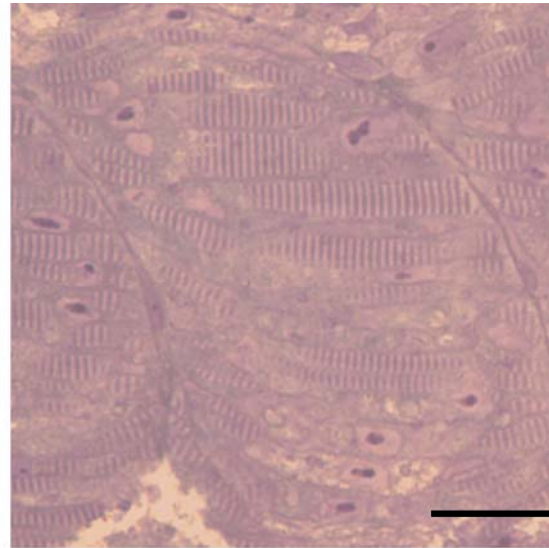

**c**

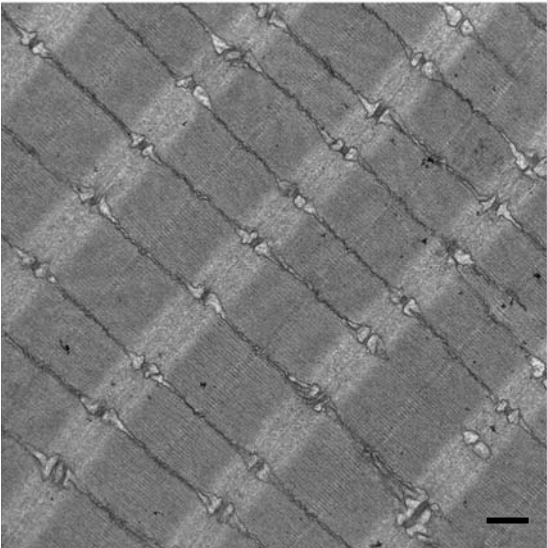

**d**

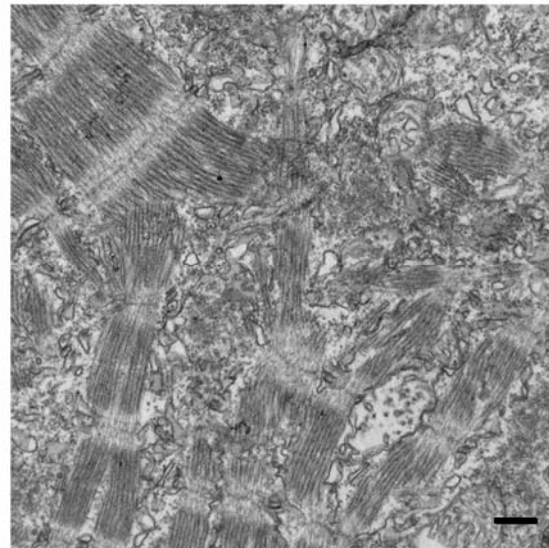

Supplement: Supplementary file 15 — Myopathic features of megf10 morphant zebrafish myofibrils a Epon-embedded semi-thin muscle sections from zebrafish injected with 6 ng of COMO were stained with toluidine blue and observed by light microscopy at 4 days post-fertilization to have striated, elongated fibers. b Similarly prepared zebrafish injected with 6 ng of MO2 showed fewer striated fibers than COMO-injected fish, and several round fibers with prominent nuclei. Scale bar for a and b is 20 μm. c Electron microscopy (EM) of ultra-thin sections showed patterned myofibrils in COMO-injected zebrafish muscle at 4 days post-fertilization. d In EM of MO2-injected zebrafish muscle, myofibrils were largely disorganized. Scale bar for c and d is 0.5 μm. (PDF 158 kb) [file 10048_2012_315_MOESM15_ESM.pdf]
