# Supplementary material for: Mutations in the satellite cell gene MEGF10 cause a recessive congenital myopathy with minicores
Source: Neurogenetics. 2012 Feb 28;13(2):115–24. doi: 10.1007/s10048-012-0315-z (PMC3332380; doi:10.1007/s10048-012-0315-z)
Supplement: Supplementary file 3 — (PDF 12 kb) [file 10048_2012_315_MOESM3_ESM.pdf]

Online Resource 3. Intervals achieving a positive LOD score in genomewide linkage scan of family 1030

Peak	Chromosome	Left flanking marker	Right flanking marker	Left boundary location	Right boundary location	Maximum LOD score
1	3	pter	rs1391931	1	2935083	1.321
2	3	rs727476	rs1995758	60316417	62091142	1.329
3	5	rs727432	rs294479	7663078	58848981	1.329
4	5	rs1490996	rs2116800	124946532	145795417	1.329
5	6	rs4110877	qter	151446716	171115067	1.329
6	7	rs1387191	rs1599405	154401017	155687803	0.01
7	8	rs1823668	rs950043	1143494	5268368	1.329
8	8	rs1074411	rs1499364	128450221	129179926	0.42
9	11	rs2349230	rs939038	25680015	33976053	1.329
10	11	rs1404501	rs2077955	63284510	72209060	0.429
11	11	rs1216518	rs2078440	100359231	123797894	1.329
12	12	pter	rs727864	1	3416399	0.11
13	13	rs726051	rs1927724	39702998	99992312	1.329
14	19	pter	rs1384936	1	5423643	0.199
15	19	rs10518254	rs739453	16022000	36079936	1.329
16	20	rs2427077	qter	59893382	63025520	0.084

All physical positions for flanking SNPs are given in NCBI Build 37 coordinates. The 16 linked regions collectively spanned 235.5 Mb of the genome.
