# Supplementary material for: Mutations in the satellite cell gene MEGF10 cause a recessive congenital myopathy with minicores
Source: Neurogenetics. 2012 Feb 28;13(2):115–24. doi: 10.1007/s10048-012-0315-z (PMC3332380; doi:10.1007/s10048-012-0315-z)
Supplement: Supplementary file 4 — (PDF 11 kb) [file 10048_2012_315_MOESM4_ESM.pdf]

Online Resource 4. Summary statistics of whole genome sequencing of subject 1030-4

#SOFTWARE_VERSION	1.8.0.23
#FORMAT_VERSION	1.3
#GENOME_REFERENCE	NCBI build 37
#DBSNP_BUILD	dbSNP build 131
#GENE_ANNOTATIONS	NCBI build 37.1

Gross mapping yield (Gb)	224.4
SNP Transitions/transversions	2.14172
SNP het/hom ratio	1.576893479
INS het/hom ratio	1.319306157
DEL het/hom ratio	1.656088404
SUB het/hom ratio	1.733661457
SNP total count	3330312
INS total count	199202
DEL total count	208476
SUB total count	70735
SNP novel rate	0.0441932
INS novel rate	0.193015
DEL novel rate	0.23182
SUB novel rate	0.303273
Fully called genome fraction	0.958889554
Partially called genome fraction	0.006667406
No-called genome fraction	0.03444304
Synonymous SNP loci	9614
Missense SNP loci	9320
Nonsense SNP loci	85
Nonstop SNP loci	18
Frame-shifting INS loci	102
Frame-shifting DEL loci	97
Frame-shifting SUB loci	24
Frame-preserving INS loci	96
Frame-preserving DEL loci	107
Frame-preserving SUB loci	262
Frame-shifting/preserving ratio	0.479569892
Nonsyn/syn SNP ratio	0.969419596
Insertion/deletions ratio	0.955515263
Ins+del/SNP ratio	0.122414356
Coding insertion/deletions ratio	0.946078431
Coding SNP/all SNP ratio	0.006226744
Coding (ins+del)/all (ins+del) ratio	0.000973808

Analysis parameters and summary statistics were provided by Complete Genomics, Inc.
