# Supplementary material for: Mutations in the satellite cell gene MEGF10 cause a recessive congenital myopathy with minicores
Source: Neurogenetics. 2012 Feb 28;13(2):115–24. doi: 10.1007/s10048-012-0315-z (PMC3332380; doi:10.1007/s10048-012-0315-z)
Supplement: Supplementary file 5 — (PDF 8 kb) [file 10048_2012_315_MOESM5_ESM.pdf]

Online Resource 5. Sequences of morpholinos injected into zebrafish embryos

MO1_i4e5	ACACGCTGCACAAAGACACAAAGCT
MO2_i5e6	CACAGCTGCACAGAGACACCAAAAC
MO3_e8i8	TATTTGCAGTGTTTGTCTCACCTGC
MO4_e17i17	AAAGCTCTTTTCCATACTGACTCGT
COMO	CCTCTTACCTCAGTTACAATTTATA

Morpholino designations are as explained in text.
